# Data mining: The association of 2‐h postprandial plasma glucose with the fasting plasma glucose in a large Chinese population

**DOI:** 10.1002/jcla.23404

**Published:** 2020-06-02

**Authors:** Dandan Sun, Dandan Li, Songlin Yu, Kai Zhang, Xinqi Cheng

**Affiliations:** ^1^ Department of Laboratory Medicine Peking Union Medical College Hospital Peking Union Medical College and Chinese Academy of Medical Science Beijing China

**Keywords:** 2‐h postprandial plasma glucose, 2‐h oral glucose tolerance test, fasting plasma glucose, insulin sensitivity, β‐cell function

## Abstract

**Background:**

It is generally believed that the lower limit of postprandial plasma glucose is the same or higher than that of fasting plasma glucose (FPG). This study aimed to investigate the relationship between 2‐h postprandial plasma glucose (2‐hPG) and FPG. Insulin sensitivity and β‐cell function were also evaluated.

**Methods:**

Analytical data from January 2013 to August 2018 included 10 465 participants’ 2‐h OGTT results and 19 518 participants’ FPG and 2‐hPG values after autonomous self‐feeding. Participants were divided into two groups based on the relationship between FPG and 2‐hPG (OGTT‐A1/Postprandial‐B1:FPG > 2‐hPG;OGTT‐A2/Postprandial‐B2:FPG ≤ 2‐hPG).Insulin sensitivity was evaluated by Matsuda index and homeostasis model assessment of insulin resistance (HOMA‐IR). β‐cell function was estimated by homeostasis model assessment of β‐cell function (HOMA‐β) and early‐phase insulin secretion index (ΔI30/ΔG30).

**Results:**

The ratio of OGTT‐A1 and OGTT‐A2 is 11.1%; the ratio of postprandial B1 and postprandial B2 is 13.7%. HOMA‐IR and HOMA‐β values were lower, while Matsuda index and ΔI30/ΔG30 values were higher in the non‐diabetic OGTT‐A1 group than those in the OGTT‐A2 group. The value of Matsuda index in women was 0.368 times higher than that in men in group OGTT‐A1. In group OGTT‐A2, the values of HOMA‐IR (0.346), HOMA‐β (9.096), and ΔI30/ΔG30 (3.575) in women were lower, higher, and higher than those in men, respectively. Both HOMA‐β and ΔI30/ΔG30 decreased with age in OGTT groups.

**Conclusion:**

It existed that FPG was >2‐hPG, and this group had better insulin sensitive and β‐cell function. The influence of age on insulin sensitivity and β‐cell function was greater than that of gender.

## 1 INTRODUCTION

Diabetes mellitus (DM) is a global epidemic. Along with population aging, urbanization, positive family history, and obesity crowdsourcing, the prevalence of diabetes continues to increase, especially in developing countries. A cross‐sectional survey that was conducted in 2013 in China revealed that China has 10.9% overall prevalence of diabetes and 35.7% prediabetic stage.^1^ China has become the country with the highest population of people with diabetes in the world.^2^

Diabetes is a common metabolic disorder characterized by hyperglycemia. Normally, the maintenance of blood glucose homeostasis depends on hormonal regulation and neuromodulation. However, hyperglycemia may result, when genetic and environmental factors contribute to the disorder of hormonal regulation and neuromodulation. Previous studies have shown a hyperbolic relationship of hyperglycemia with insulin resistance and beta‐cell dysfunction.^3^, ^4^

Several surrogate indices using glucose and insulin levels have been devised as alternative measures of insulin sensitivity, including the homeostasis model assessment of insulin resistance (HOMA‐IR) and Matsuda index.^5^, ^6^ HOMA‐IR is a model that incorporates both fasting insulin and glucose levels.^7^ Matsuda index is a model that uses dynamic glucose and insulin values obtained during oral glucose tolerance tests (OGTT).^8^

The fasting plasma glucose (FPG) and 2‐h postprandial plasma glucose (2‐hPG) levels are of great significance in the diagnosis of DM.^9^ Correct interpretation of the dynamic changes of plasma glucose is very important for clinical diagnosis and treatment. As we know, the normal FPG level is 3.9‐6.1 mmol/L. After glucose loading, the plasma glucose rises, reaching its peak in about 30‐60 minutes and then declines; it approaches the baseline level in 2 h (2‐h postprandial plasma glucose < 7.8 mmol/L).

What is the relationship between FPG and 2‐hPG in clinical practice? Must 2‐hPG be greater than FPG? To our knowledge, there are no reported studies examining this relationship using large clinical data. In this study, we investigated the relationship between FPG and 2‐hPG, in a large Chinese population. In addition, we assessed the insulin sensitivity and beta‐cell function in different groups.

## 2 MATERIALS AND METHODS

### 2.1 Data collection

**Figure 1:**
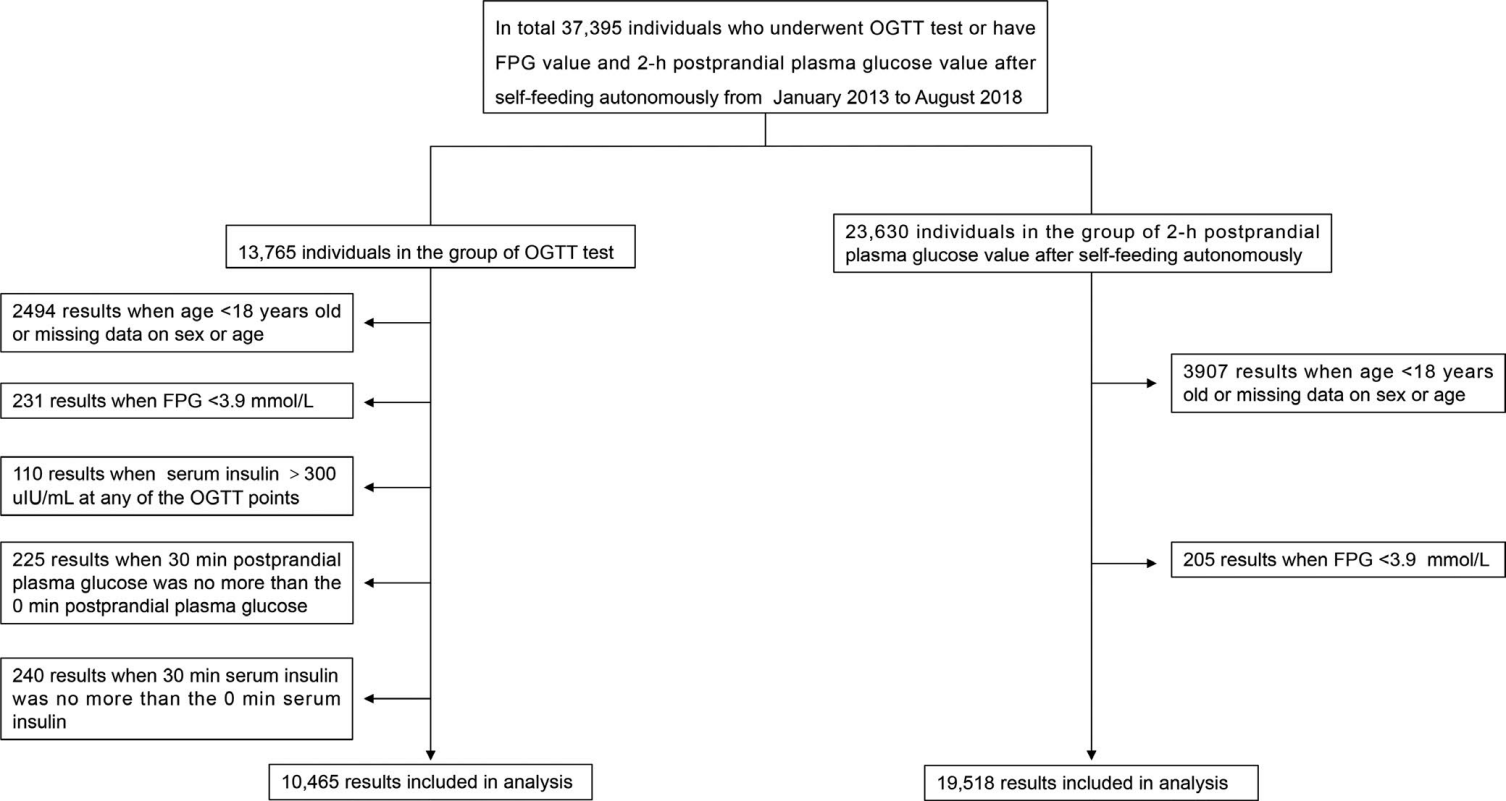
A schematic used for screening and inclusion of study samples

There are two research cohorts in this study:

In the first research cohort, analysis was based on the 2‐h OGTT data and the complete insulin data between January 2013 and August 2018 from Peking Union Medical College Hospital. Data were excluded based on the following criteria: (a) age < 18 years; (b) missing data on sex or age; (c) FPG < 3.9 mmol/L; (d) serum insulin levels > 300 μL U/mL at any of the OGTT points; (e) 30 minutes postprandial plasma glucose was no more than the 0 minutes postprandial plasma glucose; and (f) 30 minutes serum insulin was no more than the 0 minutes serum insulin. Overall, 10 465 participants’ 2‐h OGTT data were included in this study (Figure 1).

In the second research cohort, analysis was based on FPG value and 2‐hPG value with autonomous self‐feeding between January 2013 and August 2018 from Peking Union Medical College Hospital. Data were excluded based on the following: (a) age < 18 years; (b) missing data on sex or age; and (c) FPG < 3.9 mmol/L. Overall, 19 518 participants’ FPG value and 2‐hPG value after self‐feeding autonomously were included in this study (Figure 1).

Data were analyzed after removal of all personal identification information. This study was approved by the Ethics Committee of Peking Union Medical College Hospital of the Chinese Academy of Medical Sciences. As this study was retrospective in nature and the results were not traceable back to individual patients, the need for informed consent was waived.

In the first research cohort, the participants were divided into two groups (OGTT groups A1 and A2) according to the FPG and the 2‐hPG PG value (FPG value > 2‐hPG value and. FPG value ≤ 2‐hPG PG value). In the second research cohort, postprandial B1 was identified as FPG value > 2‐hPG PG value. Postprandial B2 was identified as FPG value ≤ 2‐hPG value.

### 2.2 OGTT

After an overnight fast, participants were given a solution containing the standard 75 g glucose, and venous blood samples were drawn at 0, 30, 60, 90, and 120 minutes for measuring plasma glucose and serum insulin. According to the World Health Organization criteria, diabetes was diagnosed at FPG ≥ 7.0 mmol/L and/or 2‐hPG ≥ 11.1 mmol/ L.^10^, ^11^

### 2.3 Laboratory determinations

Plasma glucose was assessed by enzymatic hexokinase photometric assay using Beckman AU2700 analyzer (Beckman Coulter). Serum insulin was assessed using Siemens ADIVA Centaur XP chemiluminescence immunoassay analyzer (Siemens Healthcare Diagnostics Inc). All analyses were standardized.

### 2.4 Calculations

The trapezoidal rule was used to calculate glucose and insulin areas under the curve during the OGTT.^12^ Insulin sensitivity was evaluated by Matsuda index and homeostasis model assessment of insulin resistance (HOMA‐IR). β‐cell function was estimated by homeostasis model assessment of β‐cell function (HOMA‐β), early‐phase insulin secretion index (ΔI30/ΔG30), the peak time frequency of insulin secretion, and insulin increment. The formulae are as follows:

1. HOMA‐IR = FPG (mmol/L) × fasting serum insulin (mU/L)/22.5.
2. HOMA‐β = 20 × fasting serum insulin (mU/L)/[fasting plasma glucose (mmol/L) − 3.5.^7^
3. Insulinogenic index = [30‐minutes serum insulin (mU/L) − fasting serum insulin (mU/L)]/[30‐minutes plasma glucose (mmol/L) − FPG (mmol/L)].^13^
4. Matsuda index = 10 000/[FPG (mg/dL) × fasting serum insulin (mU/L) × mean OGTT glucose (mg/dL) × mean OGTT insulin (mU/L)]^1/2^.^8^

### 2.5 Statistical analysis

The Kolmogorov‐Smirnov test was used to estimate data distribution. Variables with a skewed distribution were presented as medians (interquartile ranges). The Mann‐Whitney *U* test was used to determine the significance between groups. Chi‐squared test was used to compare the counting data between groups. Linear regression analysis was used to explore the effect of sex and age on insulin sensitivity and β‐cell function. The quoted P values were two‐sided, and a *P* value < .05 was considered statistically significant. All statistical analyses were performed using SPSS 20.0 (SPSS Inc).

## 3 RESULTS

### 3.1 The basic information of the involved participants

**Figure 2:**
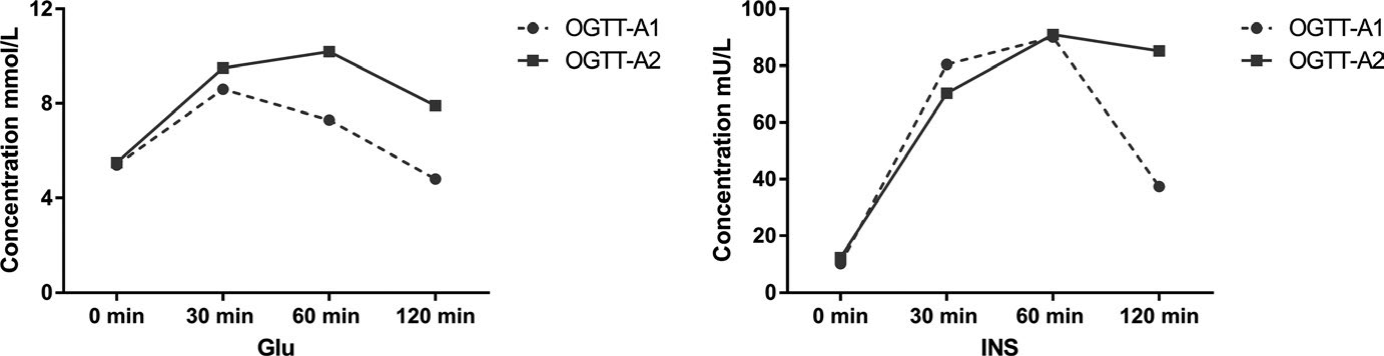
Concentration of glucose and insulin in the OGTT test. Glu, glucose; OGTT, oral glucose tolerance test; INS, insulin

As shown in Table 1, the study included a total of 10 465 participants’ 2‐h OGTT data. Of this, OGTT‐A1 and OGTT‐A2 groups had 1047 and 9418 participants’ data, respectively. The ratio of OGTT‐A1 and OGTT‐A2 is 11.1%. There are 2358 and 17 160 participants in the postprandial group, and the ratio of postprandial B1 and postprandial B2 is 13.7%. The participants are younger in OGTT‐A1 and postprandial B1 (*P* < .001). The results in Table 2 and Figure 2 showed that the glucose level of OGTT‐A1 rose to a maximum of 8.6 mmol/L at 30 minutes and then gradually decreased. After 2 hours, the postprandial plasma glucose was lower than the FPG (4.80 vs 5.40 mmol/L, respectively). However, the glucose level in the OGTT‐A2 group rose to the highest value of 10.20 mmol/L at 60 minute, and postprandial plasma glucose value was higher than for FPG (7.90 vs 5.50 mmol/L, respectively). The plasma glucose levels in OGTT‐A2 group at the four time points were higher than those of the OGTT‐A1 group (all *P* < .001). The plasma glucose levels in postprandial B2 were higher than postprandial‐B1 at fasting glucose and 2‐h glucose (both *P* < .001). The concentration of insulin level at different times in OGTT test also showed in Table 2 and Figure 2.

**Table 1:** Baseline characteristics of involved participants in the OGTT‐A and postprandial B cohort

	OGTT‐A		*P* value^a^	Postprandial B		*P* value^b^
	OGTT‐A1	OGTT‐A2		Postprandial B1	Postprandial B2	
N	1047	9418		2358	17 160	
Sex (M/F)	318/729	2536/6882	.018	435/1923	5503/11657	<.001
Age (years)	34.00 (27.00, 47.00)	37.00 (29.00, 51.00)	<.001	34.00 (27.00, 47.00)	37.00 (29.00, 51.00)	<.001

Note

OGTT‐A1: FPG > 2‐hPG; OGTT‐A2: FPG ≤ 2‐hPG; postprandial B1: FPG > 2‐hPG; postprandial B2: FPG ≤ 2‐ hPG.

Abbreviations: F, female; M, male; OGTT, oral glucose tolerance test.

^a^ The difference between OGTT‐A1 and OGTT‐A2.

^b^ The difference between postprandial B1 and postprandial B2.

**Table 2:** The glucose and insulin levels of involved participants in the OGTT‐A and postprandial B cohort

	OGTT‐A		*P* value^a^	Postprandial B		*P* value^b^
	OGTT‐A1	OGTT‐A2		Postprandial B1	Postprandial B2	
Fasting glucose (mmol/L)	5.40 (5.00, 5.90)	5.50 (5.00, 6.20)	<.001	5.30 (5.00, 6.00)	5.90 (5.10, 8.00)	<.001
30 min glucose (mmol/L)	8.60 (7.50, 9.80)	9.50 (8.30, 11.00)	<.001	‐	‐	‐
60 min glucose (mmol/L)	7.30 (5.90, 9.30)	10.20 (8.00, 10.80)	<.001	‐	‐	‐
2‐h glucose (mmol/L)	4.80 (4.30, 5.30)	7.90 (6.50, 10.60)	<.001	4.90 (4.40, 5.50)	8.40 (6.30, 13.30)	<.001
Fasting insulin (mU/L)	10.17 (6.78, 14.49)	12.41 (8.40, 17.80)	<.001	10.51 (7.10, 16.44)	11.04 (7.07, 16.61)	.392
30 min insulin (mU/L)	80.47 (50.74, 134.03)	70.27 (43.78, 123.61)	<.001	‐	‐	‐
60 min insulin (mU/L)	90.15 (53.77, 160.35)	91.04 (55.64, 154.42)	.846	‐	‐	‐
2‐h insulin (mU/L)	37.39 (22.63, 56.19)	85.17 (52.64, 151.47)	<.001	27.91 (16.21, 45.32)	49.22 (29.30, 83.99)	<.001

^a^ The difference between OGTT‐A1 and OGTT‐A2.

^b^ The difference between postprandial B1 and postprandial B2.

**Figure 3:**
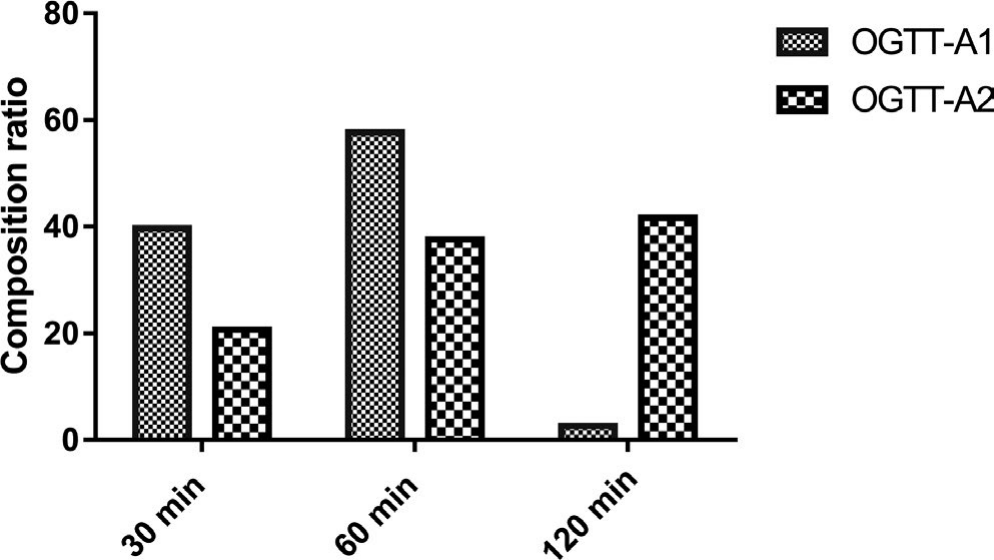
Composition ratio of insulin peaked at different time points in the OGTT experiment

From Table 3 and Figure 3, there are 57.7% participants with the highest insulin level at 60 minutes in the OGTT‐A1 group but most people reached the peak insulin level at 120 minutes in the OGTT‐A2 (41.7%).

**Table 3:** Insulin composition ratio peaked at different time points in the OGTT experiment

group	n	30 min (%)	60 min (%)	120 min (%)	*P* value
OGTT‐A1	1073	426 (39.7)	619 (57.7)	28 (2.6)	<.001
OGTT‐A2	9546	1978 (20.7)	3593 (37.6)	3975 (41.7)	

* *P* < .001, the insulin composition ratio difference between OGTT‐A1 and OGTT‐A2.

### 3.2 The insulin sensitivity and β‐cell function in the DM and non‐DM groups

To further assess the difference in insulin sensitivity and β‐cell function between the OGTT‐A1 and OGTT‐A2 groups, the participants were grouped according to whether they had diabetes or not. As shown in Table 4, in diabetic groups, there were more people in OGTT‐A2 than in OGTT‐A1. The ratio of OGTT‐A1 and OGTT‐A2 was 1.9%; however, in non‐diabetic groups, the ratio of OGTT‐A1 and OGTT‐A2 was 14.1%. The diabetic patients in both OGTT‐A1 and OGTT‐A2 groups had higher plasma glucose levels at the four time points than for non‐diabetic patients. Furthermore, the fasting insulin levels were higher in diabetic patients than in non‐diabetic patients, while non‐diabetic patients had higher insulin levels than the diabetics at 30 and 60 minutes. HOMA‐β and ΔI30/ΔG30 reflected the β‐cell function. The values of HOMA‐β, ΔI30/ΔG30, and Matsuda index were higher in the non‐diabetic group than in the diabetic group, but the value of HOMA‐IR was higher in the diabetic group than in the non‐diabetic group. The value of HOMA‐β in OGTT‐A2 was higher than that in OGTT‐A1 (all; *P* < .05), and the value of ΔI30/ΔG30 in OGTT‐A2 was lower than for OGTT‐A1 in both diabetic and non‐diabetic groups (all; *P* < .05). The level of HOMA‐IR in OGTT‐A2 (3.00) was significantly higher than that for OGTT‐A1 (2.50) in non‐diabetic patients (*P* < .001). The value of Matsuda index in OGTT‐A2 (2.84) was lower than the value of OGTT‐A1 (3.90) in non‐diabetic patients (*P* < .001).

**Table 4:** Insulin sensitivity and β‐cell function in DM and non‐DM groups based on the OGTT‐A1 and OGTT‐A2 groups

	DM		*P* value^a^	non‐DM		*P* value^b^
	OGTT‐A1	OGTT‐A2		OGTT‐A1	OGTT‐A2	
n	44	2299	<.001	1003	7119	<.001^c^
Sex (M/F)	15/29	925/1374	.410	303/700	1611/5508	<.001
Age (years)	52.50 (37.25, 60.25)	48.00 (35.00, 57.00)	.121	33.00 (27.00, 46.00)	34.00 (28.00, 47.00)	.004
Fasting glucose (mmol/L)	7.30 (7.10, 7.78)	7.00 (6.20, 7.90)	.002	5.40 (5.00, 5.80)	5.30 (4.90, 5.70)	<.001
30 min glucose (mmol/L)	11.50 (10.53, 12.56)	12.00 (10.70, 13.50)	.223	8.50 (7.40, 9.60)	8.90 (7.90.10.10)	<.001
60 min glucose (mmol/L)	11.70 (10.43, 13.38)	14.70 (13.10, 16.70)	<.001	7.20 (5.80, 9.10)	9.10 (7.50, 10.80)	<.001
2‐h glucose (mmol/L)	6.70 (6.10, 7.00)	13.70 (11.90, 16.40)	<.001	4.80 (4.30, 5.20)	7.20 (6.20, 8.50)	<.001
Fasting insulin (mU/L)	12.45 (6.77, 16.22)	12.83 (8.72, 19.17)	.301	10.12 (6.78, 14.39)	12.33 (8.30, 17.41)	<.001
30 min insulin (mU/L)	50.32 (30.35, 68.61)	42.41 (25.06, 66.25)	.147	82.36 (52.12, 137.23)	83.21 (53.46, 140.78)	.532
60 min insulin (mU/L)	87.05 (49.09, 168.46)	63.79 (39.49‐114.80)	.010	90.15 (53.78, 160.11)	102.20 (62.08, 164.11)	<.001
2‐h insulin (mU/L)	48.46 (30.13, 72.05)	82.15 (47.34, 152.67)	<.001	37.19 (22.37, 56.03)	85.98 (54.33, 151.11)	<.001
HOMA‐β	61.20 (31.58, 87.13)	76.90 (46.10, 123.70)	.026	108.80 (74.00, 162.80)	136.60 (92.70, 206.50)	<.001
HOMA‐IR	4.30 (2.33, 7.23)	4.20 (2.70, 6.50)	.760	2.50 (1.60, 3.60)	3.00 (2.00, 4.30)	<.001
ΔI30/ΔG30	10.30 (5.25, 16.90)	5.60 (3.10, 10.00)	<.001	25.30 (13.80, 45.60)	20.50 (11.60, 35.50)	<.001
Matsuda index	2.68 (1.82, 4.53)	2.29 (1.53, 3.47)	.096	3.90 (2.70, 5.66)	2.84 (1.97, 4.23)	<.001

^a^ The difference between OGTT‐A1 and OGTT‐A2 in the DM.

^b^ The difference between OGTT‐A1 and OGTT‐A2 in the non‐DM.

^c^ The difference between DM and non‐DM.

### 3.3 Effect of sex and age on insulin sensitivity and β‐cell function

The results in Table 4 showed that there existed significant difference of HOMA‐β and ΔI30/ΔG30 between OGTT‐A1 and OGTT‐A2 groups (all *P* < .001). In the Table 1, it is easy to find that the sex and age are different between OGTT‐A1 and OGTT‐A2 groups (Both < 0.05). Therefore, linear regression analysis was used to explore whether the sex and age may have effect on insulin sensitivity and β‐cell function. The results were shown in Table S1.

There were no significant differences between men and women in HOMA‐β, HOMA‐IR, and ΔI30/ΔG30 in OGTT‐A1 (all; *P* > .05). However, the Matsuda index in women was 0.368 higher than for men in OGTT‐A. In group OGTT‐A2, the values of HOMA‐β (9.096) and ΔI30/ΔG30 (3.575) were higher while that of HOMA‐IR (0.346) were lower in women, than the values in men (all; *P* < .001).

The level of HOMA‐β in those aged 30‐39, 40‐49, 50‐59, and ≥60 years decreased gradually compared to those in the age group 18‐29 years, for both OGTT groups. The level of HOMA‐β in those aged ≥ 60 years for OGTT‐A1 and OGTT‐A2 was 100.146 and 107.055 lower than the group 18‐29 years, respectively. The level of HOMA‐IR in those aged ≥ 60 years was 1.177 and 1.100 for OGTT‐A1 and OGTT‐A2, respectively, and lower than the level for those aged 18‐29 years. HOMA‐β and HOMA‐IR decreased with age in the OGTT‐A2 group. The level of ΔI30/ΔG30 in those aged 30‐39, 40‐49, 50‐59 and ≥60 decreased compared to those aged 18‐29 years in both groups with little fluctuations. However, the decrease in ΔI30/ΔG30 with age in the OGTT‐A2 group was smaller than that in the OGTT‐A1 group. The Matsuda index increased with age in both groups (all *P* < .001); but the degree of increase in OGTT‐A1 was greater than that in the OGTT‐A2 group.

According to the standard beta coefficient in Table S1, the influence of age on HOMA‐β, HOMA‐IR, ΔI30/ΔG30, and Matsuda index was greater than that of gender.

## 4 DISCUSSION

This study investigated the proportion of patients with postprandial blood glucose level below the FPG level and evaluated insulin sensitivity and β‐cell function, based on the relationship between FPG and 2‐hPG in a large representative population covering all adult age groups in China.

Our data showed that the level of 2‐hPG was lower than the FPG in clinical practice, but the composition ratio was relatively small. The ratio of OGTT‐A1 and OGTT‐A 2 is 11.1%, and the ratio of postprandial B1 and postprandial B2 is 13.7%. However, in the DM and non‐DM groups, the ratio of the OGTT‐A1 and OGTT‐A2 group was approximately 1.9% and 14.1%, respectively. This implies fewer number of diabetic patients in whom the 2‐h postprandial plasma glucose value was less than the FPG compared to those in whom the 2‐h postprandial plasma glucose level was greater than the FPG after OGTT.

From our data analysis results, the fasting insulin and 2‐h insulin levels of OGTT‐A2 group were higher than those of the OGTT‐A1 group. In addition, more people in the OGTT‐A1 group reached the peak of insulin secretion at 60 minute, but large number of people in the OGTT‐A2 group reached the peak of insulin secretion at 120 minutes in the diabetic patients. Studies have found that insulin secretion delay is associated with decreased insulin sensitivity, early insulin response, and increased postprandial blood glucose.^14^, ^15^

HOMA‐IR was lower in the OGTT‐A1 group than in the OGTT‐A2 group in the non‐diabetic patients, and the value of Matsuda index was greater in the OGTT‐A1 group than in the OGTT‐A2 group, indicating that the insulin sensitivity of the OGTT‐A1 group was stronger than that of the OGTT‐A2 group. However, the values of HOMA‐IR and Matsuda index were not significantly different between the two groups in diabetic patients. Early loss of the first‐phase secretion has long been considered a hallmark of diabetes, and ΔI30/ΔG30 is a good indicator of early secretory function.^16^ We also evaluated the function of islet β cells by HOMA‐β and ΔI30/ΔG30. The results showed a lower HOMA‐β values in the OGTT‐A1 group than those in the OGTT‐A2 group. This finding might be because the OGTT‐A2 group had stronger insulin resistance, and thus, the islet β cells function was compensating. The value of ΔI30/ΔG30 was higher in the OGTT‐A1 group than in the OGTT‐A2 group, indicating that the islet β cell function of the OGTT‐A1 group was stronger than that in the OGTT‐A2 group.

Overall, there existed the situation that the participants with FPG value > 2‐hPG value, although the ratio was small, it was not the wrong results and the insulin resistance of the OGTT‐A1 group was weaker than that of the OGTT‐A2 group, and the β‐cell function of the OGTT‐A1 group was stronger than that of the OGTT‐A2 group. Therefore, clinicians should pay full attention to these two situations. The conclusion of our study may provide a reference value for monitoring the progress of the patient's condition and make sure that there exists difference of the β cells function and insulin resistance between OGTT‐A1 and OGTT‐A2 groups. It is important to clarify the functional status of individual β cells and the degree of insulin resistance for clinical effective intervention in abnormal glucose metabolism. More indicators evaluating beta‐cell function and insulin sensitivity need to be tested for the OGTT‐A2 group,^17^ and clinicians should pay attention to the patients with FPG value ≤ 2‐hPG value and take preventive strategies or interventions timely.

Researcher lay less emphasis on the effect of gender and age for insulin resistance and beta‐cell function.^18^ From the Table S1, although there are no significance between men and women in HOMA‐β, HOMA‐IR, and ΔI30/ΔG30 in group OGTT‐A1, the value of Matsuda index in women was 0.368 higher than in men in group OGTT‐A1. The value of HOMA‐β in women was 9.096 higher than in men in group OGTT‐A2. Similarly, the value ofΔI30/ΔG30 in women was 3.575 higher than in men in group OGTT‐A2. The value of HOMA‐IR in women was 0.346 lower than in men in group OGTT‐A2. It is easy to find that women's insulin resistance is weaker than that of men, and women's islet B cells function is better than men.

Both HOMA‐β and ΔI30/ΔG30 decreased with age in both OGTT‐A1 and OGTT‐A2 groups, indicating that islet function is getting worse with age. Insulin sensitivity was evaluated by Matsuda index and HOMA‐IR. HOMA‐IR has a downward trend with increasing age, but the Matsuda index has an increasing trend with age. The sensitivity of the Matsuda index is better. Similar to our study, Veeradej Pisprasert et al^19^ found Matsuda index was superior to HOMA‐IR in overall African Americans subgroup consisting of males and females. Matsuda index appeared to be most reliable in studies of African American. Our results indicated that the influence of age on HOMA‐β, HOMA‐IR, ΔI30/ΔG30, and Matsuda index was greater than that of gender. In future studies, researchers should examine for the effect of gender and age on insulin resistance and beta‐cell function.

The present study was retrospective in design and based on a big clinical data, with several advantages. First, this study compared the differences in insulin resistance and β‐cell function between the OGTT‐A1 and OGTT‐A2 groups. In addition, this study was the first to describe the ratio of FPG value > 2‐hPG value. However, this study also has several limitations. First, we did not have detailed information on patients’ clinical diagnosis. Second, there was no basic patients’ information such as height and weight; thus, it was impossible to calculate indicators such as the body mass index or to evaluate its effects on insulin resistance and β‐cell function. Additionally, it was difficult to avoid selective bias in a big data analysis, since we only evaluated the effect of biological factors on insulin resistance and β‐cell function in this Chinese population. Therefore, in future studies, we hope to collaborate with foreign researchers to conduct this study worldwide. However, the finding of this study clearly showed a difference in insulin resistance and β‐cell function between the OGTT‐A1 and OGTT‐A2 groups; therefore, these limitations did not affect our conclusions.

## 5 CONCLUSIONS

Findings showed the ratio of OGTT‐A1 and OGTT‐A 2 is 11.1% suggest that the number of FPG > 2‐hPG level was less than the FPG value was far less than those in whom the 2‐hPG level was greater than the FPG after OGTT. Overall, the insulin resistance of the OGTT‐A1 group was weaker than that of the OGTT‐A2 group, and the β‐cell function of the OGTT‐A1 group was stronger than that of the OGTT‐A2 group. Therefore, clinicians should pay full attention to these two situations both when monitoring the progression of the patient's condition and when they need to take appropriate treatment measures.

## CONFLICT OF INTEREST

The authors declare that they have no competing interests.

## AUTHOR CONTRIBUTIONS

Dandan Sun designed the study. Dandan Sun and Dandan Li analyzed the data and wrote the manuscript. Songlin Yu and Kai Zhang contributed reagents/materials/analysis tools. Xinqi Cheng revised the draft. All authors reviewed the manuscript and approved the final manuscript.

## Supporting information

Table S1
