# Supplementary material for: Data mining: The association of 2‐h postprandial plasma glucose with the fasting plasma glucose in a large Chinese population
Source: J Clin Lab Anal. 2020 Jun 2;34(9):e23404. doi: 10.1002/jcla.23404 (PMC7521326; doi:10.1002/jcla.23404)
Supplement: Supplementary file 1 — Table S1 [file JCLA-34-e23404-s001.doc]

Supplemental Table 1. Linear regression analysis of insulin sensitivity and β-cell function by sex and age

|  | | | | | | | 95% of beta | |
| --- | --- | --- | --- | --- | --- | --- | --- | --- |
|  |  | Non-standard beta coefficient | Standard error | Standard beta coefficient | t | P | Lower | Upper |
| a. Dependent Variable: HOMA-β | | | | | | | | |
| OGTT-A1 | (Constant) | 186.856 | 11.039 |  | 16.927 | <0.001 | 165.194 | 208.517 |
|  | Sex | -5.483 | 5.687 | -.028 | -.964 | .335 | -16.642 | 5.676 |
|  | 30-39 years | -45.895 | 6.514 | -.224 | -7.046 | <0.001. | -58.677 | -33.113 |
|  | 40-49 years | -86.734 | 7.935 | -.344 | -10.930 | <0.001 | -102.304 | -71.163 |
|  | 50-59 years | -92.706 | 7.928 | -.363 | -11.694 | <0.001 | -108.263 | -77.150 |
|  | ≥60 years | -100.146 | 10.363 | -.289 | -9.663 | <0.001 | -120.481 | -79.810 |
| OGTT-A2 | (Constant) | 190.296 | 5.182 |  | 36.722 | <0.001 | 180.138 | 200.454 |
|  | Sex | 9.096 | 2.540 | .036 | 3.582 | <0.001 | 4.118 | 14.074 |
|  | 30-39 years | -51.370 | 2.928 | -.208 | -17.542 | <0.001 | -57.110 | -45.629 |
|  | 40-49 years | -84.006 | 3.548 | -.270 | -23.676 | <0.001 | -90.961 | -77.051 |
|  | 50-59 years | -100.394 | 3.506 | -.327 | -28.637 | <0.001 | -107.266 | -93.522 |
|  | ≥60 years | -107.055 | 3.862 | -.305 | -27.717 | <0.001 | -114.626 | -99.484 |
| a. Dependent Variable: HOMA-IR | | | | | | | | |
| OGTT-A1 | (Constant) | 4.050 | .401 |  | 10.111 | <0.001 | 3.264 | 4.836 |
|  | Sex | -.211 | .206 | -.032 | -1.021 | .308 | -.615 | .194 |
|  | 30-39 years | -.636 | .236 | -.094 | -2.692 | .007 | -1.100 | -.172 |
|  | 40-49 years | -1.056 | .288 | -.126 | -3.666 | <0.001 | -1.621 | -.491 |
|  | 50-59 years | -.827 | .288 | -.098 | -2.874 | .004 | -1.391 | -.262 |
|  | ≥60 years | -1.177 | .376 | -.103 | -3.131 | .002 | -1.915 | -.440 |
| OGTT-A2 | (Constant) | 5.112 | .147 |  | 34.835 | <0.001 | 4.824 | 5.399 |
|  | Sex | -.346 | .072 | -.051 | -4.809 | <0.001 | -.487 | -.205 |
|  | 30-39 years | -.568 | .083 | -.086 | -6.855 | <0.001 | -.731 | -.406 |
|  | 40-49 years | -.928 | .100 | -.112 | -9.235 | <0.001 | -1.125 | -.731 |
|  | 50-59 years | -1.010 | .099 | -.123 | -10.173 | <0.001 | -1.204 | -.815 |
|  | ≥60 years | -1.100 | .109 | -.118 | -10.057 | <0.001 | -1.314 | -.886 |
| a. Dependent Variable: ΔI30/ΔG30 | | | | | | | | |
| OGTT-A1 | (Constant) | 41.297 | 6.758 |  | 6.110 | <0.001 | 28.035 | 54.559 |
|  | Sex | 6.775 | 3.482 | .060 | 1.946 | .052 | -.057 | 13.607 |
|  | 30-39 years | -14.878 | 3.988 | -.127 | -3.731 | <0.001 | -22.704 | -7.053 |
|  | 40-49 years | -26.669 | 4.858 | -.185 | -5.489 | <0.001 | -36.202 | -17.136 |
|  | 50-59 years | -31.080 | 4.854 | -.212 | -6.403 | <0.001 | -40.604 | -21.556 |
|  | ≥60 years | -30.870 | 6.345 | -.156 | -4.866 | <0.001 | -43.320 | -18.421 |
| OGTT-A2 | (Constant) | 26.097 | 1.220 |  | 21.397 | <0.001 | 23.706 | 28.488 |
|  | Sex | 3.575 | .598 | .061 | 5.981 | <0.001 | 2.403 | 4.747 |
|  | 30-39 years | -9.013 | .689 | -.159 | -13.078 | <0.001 | -10.365 | -7.662 |
|  | 40-49 years | -15.189 | .835 | -.214 | -18.188 | <0.001 | -16.826 | -13.552 |
|  | 50-59 years | -17.381 | .825 | -.247 | -21.065 | <0.001 | -18.999 | -15.764 |
|  | ≥60 years | -16.834 | .909 | -.210 | -18.518 | <0.001 | -18.616 | -15.052 |
| a. Dependent Variable: Matsuda index | | | | | | | | |
| OGTT-A1 | (Constant) | 3.184 | .340 |  | 9.379 | <0.001 | 2.518 | 3.851 |
|  | Sex | .368 | .175 | .066 | 2.104 | .036 | .025 | .711 |
|  | 30-39 years | .669 | .200 | .115 | 3.341 | .001 | .276 | 1.063 |
|  | 40-49 years | 1.398 | .244 | .195 | 5.729 | <0.001 | .919 | 1.877 |
|  | 50-59 years | 1.142 | .244 | .158 | 4.684 | <0.001 | .664 | 1.621 |
|  | ≥60 years | .866 | .319 | .088 | 2.717 | .007 | .241 | 1.492 |
| OGTT-A2 | (Constant) | 2.920 | .101 |  | 28.853 | <0.001 | 2.721 | 3.118 |
|  | Sex | -.017 | .050 | -.004 | -.352 | .725 | -.115 | .080 |
|  | 30-39 years | .401 | .057 | .088 | 7.010 | <0.001 | .289 | .513 |
|  | 40-49 years | .631 | .069 | .111 | 9.103 | <0.001 | .495 | .767 |
|  | 50-59 years | .513 | .068 | .091 | 7.487 | <0.001 | .378 | .647 |
|  | ≥60 years | .659 | .075 | .102 | 8.733 | <0.001 | .511 | .806 |

HOMA-β, homeostasis model assessment of β-cell function; OGTT, oral glucose tolerance test; HOMA-IR, homeostasis model assessment of insulin resistance; ΔI30/ΔG30, early-phase insulin secretion index
